# *Ajuga reptans* L. Herb Extracts: Phytochemical Composition and Pharmacological Activity Screening

**DOI:** 10.3390/plants14020219

**Published:** 2025-01-14

**Authors:** Svitlana Maliuvanchuk, Andriy Grytsyk, Oksana Popadynets, Taras Kotyk, Ain Raal, Oleh Koshovyi

**Affiliations:** 1 Department of Pharmaceutical Management, Drug Technology and Pharmacognosy, Ivano-Frankivsk National Medical University, 76000 Ivano-Frankivsk, Ukraine; sv_malyv@ukr.net (S.M.); grycyk@ukr.net (A.G.); 2 Department of Anatomy, Ivano-Frankivsk National Medical University, 76000 Ivano-Frankivsk, Ukraine; opopadynets@ifnmu.edu.ua (O.P.); tkotyk@ifnmu.edu.ua (T.K.); oleh.koshovyi@ut.ee; 3 Institute of Pharmacy, Faculty of Medicine, University of Tartu, Nooruse 1, 50411 Tartu, Estonia; 4 Department of Pharmacognosy and Nutriciology, The National University of Pharmacy, 61002 Kharkiv, Ukraine

**Keywords:** *Ajuga reptans* L., extract, phenolic compounds, hepatoprotective activity, hemostatic activity, wound-healing activity

## Abstract

The genus *Ajuga* (Lamiaceae family) comprises approximately 300 species, which are widely used in traditional medicine for their diaphoretic, antiseptic, hemostatic, and anti-inflammatory properties, but scarcely in official ones. Therefore, the study of *Ajuga reptans* holds promise for developing new medicinal products. In aqueous and aqueous-alcoholic soft extracts of the *A. reptans* herb, 16 amino acids, 20 phenolics, and 10 volatile substances were identified by HPLC and GC/MS. The assays of the main substances’ groups were also determined by spectrophotometry (vitamin K1, polyphenols, tannins, flavonoids, and hydroxycinnamic acids) and titrometry (ascorbic and organic acids). *A. reptans* herb extracts are practically non-toxic, exhibit hepatoprotective activity (dose 25 mg/kg) in experimental carbon tetrachloride-induced hepatitis, moderate anti-inflammatory activity (dose 100 mg/kg) in carrageenan-induced edema models, and possess significant local hemostatic (reducing bleeding time by 40.6%) and wound-healing properties (complete wound healing after 9 days). The aqueous-ethanolic soft *A. reptans* extract (extractant 50% ethanol) demonstrated the most pronounced hepatoprotective and anti-inflammatory effects. *A. reptans* extracts are capable of inhibiting the growth of microorganisms and showing higher activity against Gram-positive bacteria. *A. reptans* herb extracts are promising agents for implementation in official medicine as wound healing and hepatoprotective remedies after further preclinical and clinical studies.

## 1. Introduction

**Figure 1:**
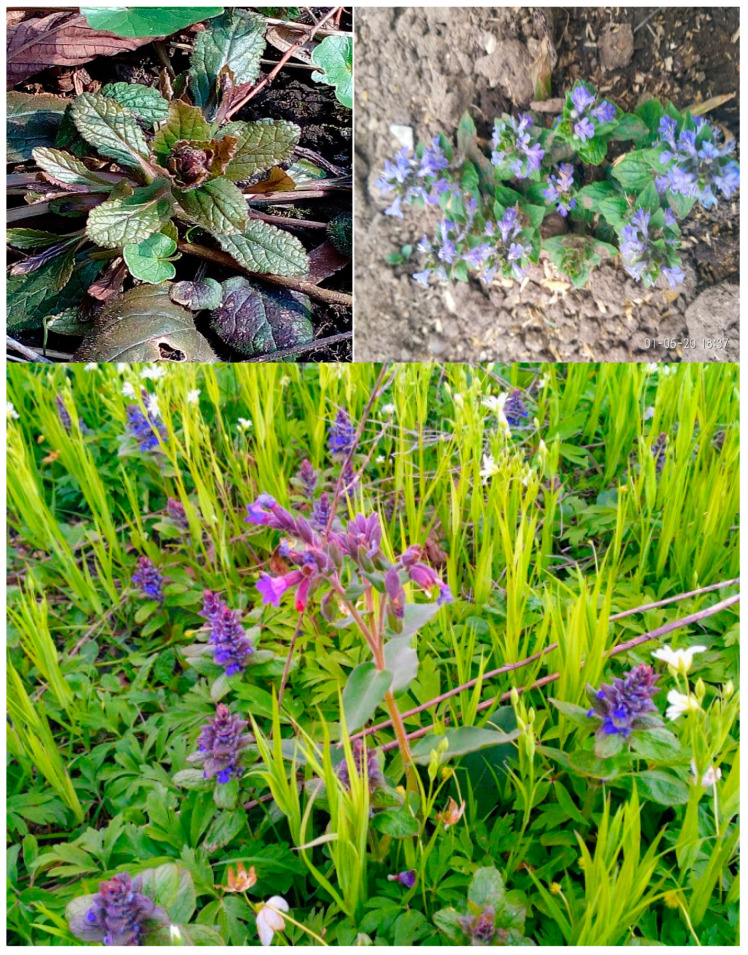
*Ajuga reptans* L.

According to the World Flora Online, the genus *Ajuga* in the Lamiaceae family includes approximately 300 species, of which 91 species are recognized as independent taxa, 192 have synonymous names, and 10 species remain undefined [1]. The International Plant Names Index lists 261 species of the genus *Ajuga* [2]. Nine species of this genus grow in Ukraine, the most common being *Ajuga reptans* L. (Figure 1), *A. genevensis* L., and *A. laxmannii* (Murray) Benth. However, plants of the genus *Ajuga* are not officially recognized in main pharmacopeias but are widely used in traditional medicine across many countries. Species of the genus *Ajuga* are used as ornamental plants due to their vibrant flower colors and prolonged blooming periods. These plants are appreciated for their varied leaf shapes, textures, and colors.

The aerial parts of *Ajuga* plants contain various biologically active substances (BAS), including flavonoids, hydroxycinnamic acids, essential oils, alkaloids, tannins, organic acids, and others [3,4]. The chemical composition of *A. reptans* is relatively understudied. The quantitative composition and antioxidant activity of ethanolic extracts from the root and leaves of *A. reptans* were carried out. The dominant compounds in both extracts were verbacoside, isoverbacoside, 3,4-dihydroxyphenylacetic acid, and rosmarinic acid [5]. This plant is rich in phenolics, so it may have significant anti-inflammatory and hepatoprotective activity. *A. reptans* raw materials also contain volatile oils, iridoids (such as 8-*O*-acetyl-harpagide) [6], flavonoids (including isoquercitrin) [7], tannins, and traces of alkaloids [8]. Phytoecdysteroids have also been isolated from *A. reptans*, including 20-hydroxyecdysone, 29-norcyasterone, 5,20-dihydroexidizone, sengosterone, ajugalactone, and ajugosterone [4]. The herb also contains macro- and microelements [9] as well as fatty acids [10].

Species of the genus *Ajuga* are recognized for their diaphoretic, antiseptic, hemostatic, and anti-inflammatory properties [4,11]. Most species of this genus are used to treat viral diseases, colds, rheumatism, stomach diseases, and gallstone disease [12]. Some species are also used in the treatment of malaria and oncology [13,14,15,16]. The herb of *A. reptans* is used in traditional Austrian medicine as a tea to treat respiratory disorders [4]. In traditional Bulgarian medicine, the plant is regarded as a remedy that enhances metabolism and is also used for gastrointestinal diseases [17]. In Polish folk medicine, *A. reptans* is used for its laxative, analgesic, and astringent properties and is known for its anti-edematous, anti-hemorrhagic, and anti-inflammatory effects [18]. *A. reptans* extracts demonstrated antiproliferative potential against prostate and lung cancer cells [7]. *Ajuga* sp. extracts have antimicrobial activity against *C. albicans*, *C. tropicalis*, and *C. parapsilosis* [19]. As *A. reptans* remedies have been successfully used in folk medicine of many countries and there is less scientific confirmation of their efficiency, it is advisable to carry out screening of some kinds of pharmacological activity mentioned in these sources. *A. reptans* is cultivated in the botanical garden of the renowned cosmetic brand Yves Rocher as a plant that enriches the “Vegetal” lifting line. In 1959, Yves Rocher’s plant cosmetics experts studied *A. reptans* and discovered its high collagen content, which has a pronounced lifting effect. The company’s scientists successfully extracted and patented a collagen concentrate, subsequently developing a new facial care line that restores skin structure using a biotechnological process [20]. Additionally, the phenylpropanoid glycoside from *A. reptans* has been used to create a composition for the prevention and treatment of androgenic alopecia and telogen effluvium [21]. *A. reptans* extracts positively affect the condition of the skin, causing an improvement in the degree of skin hydration and elasticity, reducing skin pore size and skin hyperpigmentation, and reducing wrinkle depth [22].

A literature review indicates that the genus *Ajuga* are valuable medicinal species that have been used in traditional medicine across various countries for a long time [4,6,12]. Due to the beneficial therapeutic effects of the *Ajuga* genus, it can be considered in future preclinical and clinical studies as a source of natural antioxidants [23], dietary supplements in the pharmaceutical industry, and stabilizing food against oxidative deterioration [12]. They contain a complex array of BAS and exhibit diverse pharmacological activities. However, *Ajuga* species have been scarcely studied, and the available data on their distribution and medical use suggest promising opportunities for further research and the development of new medicinal products for clinical and pharmaceutical applications. The experience of using *A. reptans* raw material in folk medicine indicates the feasibility of carrying out pharmacological activity screening, especially in antimicrobial, anti-inflammatory, hemostatic, wound healing, and hepatoprotective activity

The purpose of the study was to study the phytochemical composition of soft aqueous and aqueous-alcoholic extracts of *A. reptans* L. herb and carry out screening of their pharmacological activity to establish their potential for use in official medical and pharmaceutical practice.

## 2. Results

The soft extracts (AR) obtained from the *A. reptans* herb are viscous masses of dark brown color with a faint specific odor. The yield of extracts was as follows: AR1 (extractant water) 26.2% ± 0.18%, AR2 (50% ethanol) 28.4% ± 0.21%, and AR3 (70% ethanol) 28.9% ± 0.24%. The yields of extractive substances were calculated as the average result of three experiments.

### 2.1. Phytochemical Research

Using commonly accepted qualitative chemical reactions and chromatographic methods [24,25,26], the presence of organic acids, amino acids, phenolic compounds including hydroxycinnamic acids, coumarins, flavonoids, and tannins, iridoids, terpenoids, saponins, phytosterols, and vitamin K in the extracts of the *A. reptans* herb was confirmed. As a result of the analysis using high-performance thin-layer chromatography (HPTLC) (Figure 1), in the *A. reptans* herb extracts, 13 to 15 phenolic compounds belonging to hydroxycinnamic acids, flavonoids, and coumarins were found, but only six of them (rutin, caffeic acid, quercetin, luteolin, apigenin, and ferulic acid) were identified.

**Figure 2:**
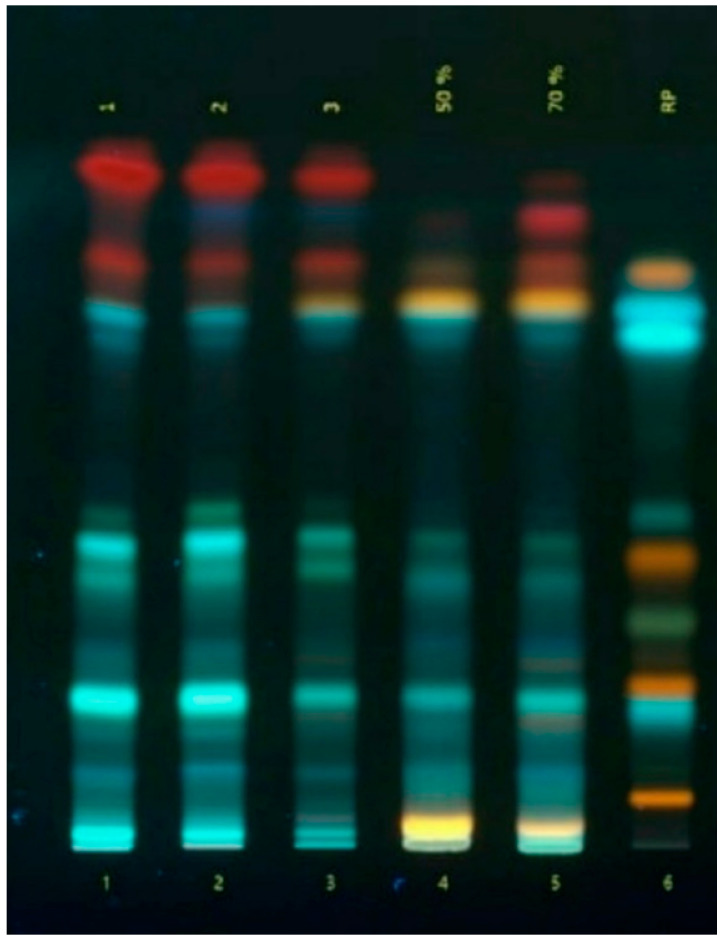
TLC chromatogram of phenolic compounds in *Ajuga reptans* L. herb extracts: 1–3—extracts of *A. reptans*; 4—*A. reptans* extract (extraction agent: 50% ethanol); 5—*A. reptans* extract (extraction agent: 70% ethanol); 6—comparison solution (rutin (R_f_ = 0.05), apigenin-7-glucoside (R_f_ = 0.07), chlorogenic acid (R_f_ = 0.22), isoquercitrin (R_f_ = 0.24), hyperoside (R_f_ = 0.26), apigenin (R_f_ = 0.35), luteolin (R_f_ = 0.42), caffeic acid (R_f_ = 0.45), ferulic acid (R_f_ = 0.77), quercetin (R_f_ = 0.79)).

The quantitative content of the main groups of BAS in the studied extracts was determined. The content of vitamin K_1_, recalculated as Vikasol, and other phenolic compounds in the extracts of the *A. reptans* herb, determined by the spectrophotometric method, are presented in Table 1 Using TLC and high-performance liquid chromatography (HPLC), 20 phenolic compounds and quinic acid were identified and quantified in the extracts of the *A. reptans* herb (Table 1, Figure 2 and Figure S1). Among phenolic compounds, there were three phenol carboxylic acids, five hydroxycinnamic acids, eight flavonoids, and four tannin metabolites. The research results presented in Table 1 indicate that among the identified hydroxycinnamic compounds, the dominant ones were *p*-coumaric and caffeic acids. Among the flavonoids, the predominant compounds were rutin and quercetin. There was also a tendency to accumulate condensed tannins, specifically their metabolites, including pyrocatechin, gallocatechin, epicatechin, and epicatechin gallate.

Using gas chromatography-mass spectrometry (GC-MS) [27,28], the qualitative composition and quantitative content of some compounds from the volatile fractions in the extracts of the *A. reptans* herb were studied (Table 2). The volatile compounds were isolated from the extracts with hexane. GC analysis was performed on all hexane fractions. The error of the GC apparatus itself is less than 1%. As the aqueous extract AR1 of the *A. reptans* herb contained traces of terpenoids, these results were not presented in Table 2.

In the volatile fraction of the *A. reptans* herb extracts, 10 compounds were identified, while hexahydrofarnesyl acetone and squalene were the dominant compounds.

The qualitative composition and quantitative content of amino acids in the extracts of the *A. reptans* herb were studied (Table 3).

Sixteen amino acids were identified, including seven essential amino acids: threonine, valine, methionine, isoleucine, leucine, phenylalanine, and lysine, as well as one conditionally essential amino acid, histidine. Glutamic acid, aspartic acid, arginine, leucine, serine, valine, and glycine have high concentrations in the extracts.

### 2.2. Pharmacological Research

#### 2.2.1. Acute Toxicity

When a single intragastric administration of the permissible doses of the studied extracts was administered, no animals died. The animals were weighed on days 1, 3, 7, and 14. Observations were conducted over 14 days, during which the extracts did not negatively affect the functioning of vital organs and systems, including the liver, kidneys, and blood. All animals were macro-necropsied. Only visual examination of organs without histopathological research has been carried out. The organs of the animals receiving the studied extracts did not differ in shape, size, color, or consistency from those of the control group. The serous tissues in the abdominal cavity remained unchanged. The obtained results indicate the absence of hepatotoxicity during the intake of *A. reptans* extracts. Therefore, the findings suggest that intragastric administration of the *A. reptans* herb extracts at a dose of 5000 mg/kg did not result in animal mortality, indicating an absence of toxic effects at this dose and characterizing the extracts as practically non-toxic (toxicity class V, LD50 > 5000 mg/kg) according to the toxicity classification of substances.

#### 2.2.2. Hepatoprotective Activity

**Figure 3:**
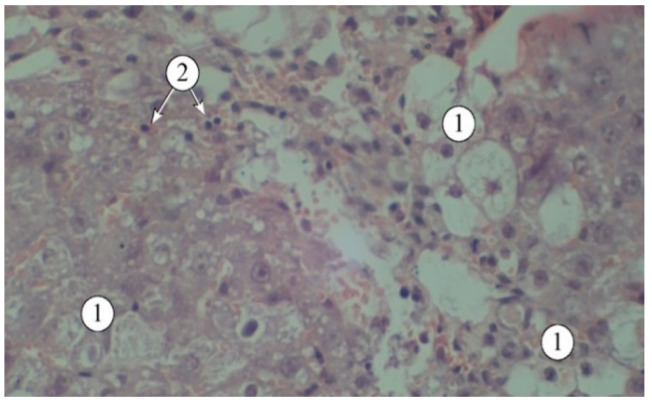
Structural features of liver lobules under the influence of the toxicant. 1—destructured hepatocytes, 2—lymphocytes. Staining: hematoxylin and eosin. Magnification: ×200.

**Figure 4:**
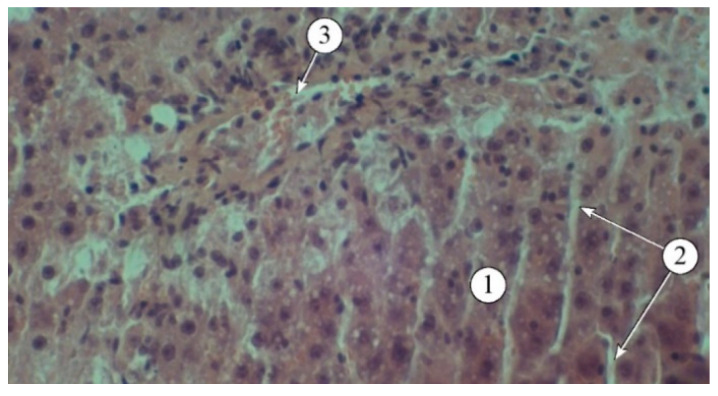
Structural features of liver lobules in animals administered AR2: 1—hepatic plates, 2—sinusoids, 3—blood vessels. Staining: hematoxylin and eosin. Magnification: ×200.

**Figure 5:**
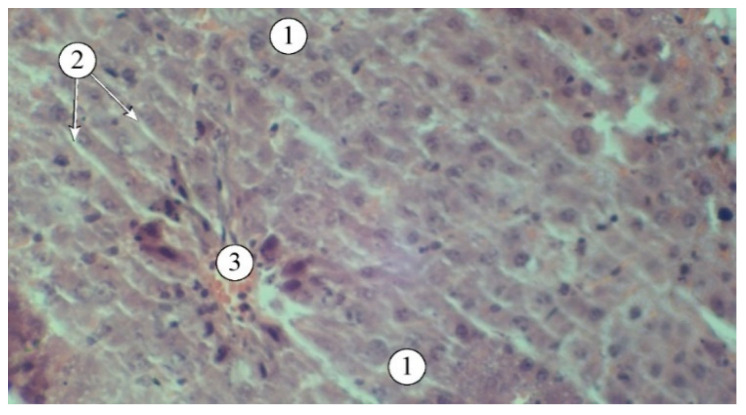
Structural features of liver lobules in animals that received the “Silibor” preparation. 1—hepatic plates, 2—sinusoid, 3—central vein. Staining: hematoxylin and eosin. Magnification: ×200.

The hepatoprotective activity of the *A. reptans* herb extracts was assessed using a model of acute carbon tetrachloride-induced hepatitis [29,30]. The results of the biochemical studies of blood serum and liver homogenate are presented in Table 4. Structural features are demonstrated in Figure 3, Figure 4 and Figure 5.

The destruction of cell membrane components (Table 4) resulted in the development of a pronounced cytolytic syndrome, as indicated by a 5.7-fold increase in ALT activity in the blood serum. The development of acute toxic hepatitis was characterized by an intensification of peroxide catabolic transformations, as evidenced by a 1.7- and 2.3-fold increase in the content of TBA-reactive substances in the blood serum and liver homogenate of the control group animals, respectively, compared to the indicators of intact animals.

The results of the studies (Figure 3) indicate that a single administration of carbon tetrachloride was accompanied by the development of acute toxic liver damage, with a loss of structural integrity, disorganized hepatic plates, and the portal triad not being visualized. The interlobular spaces were expanded and deformed, with pronounced lymphocytic infiltration. The borders of hepatocytes were blurred, and in many fields of view, the nuclei were not visible. There was a pronounced macro- and microvesicular component in the cytoplasm of hepatocytes in the peripheral zone.

The use of extracts AR1, AR2, AR3, and the comparison drug “Silibor” in the context of experimental hepatitis was associated with a noticeable reduction in pathological manifestations (Figure 4). The best results were observed in animals that received AR2; the histostructure of the liver was characterized by the organization of hepatic plates and visualization of the components of the portal triad. Hepatocytes in the peripheral zone had a polygonal shape, with their cytoplasm filled with fine-dispersed content and uniformly filled with small vesicles. The intermediate and central zones of the liver lobule demonstrated structured hepatocytes and sinusoids.

In the liver of animals that received the “Silibor” preparation, light-optical examination revealed hepatic plates that were radially oriented towards the central vein and composed of two rows of hepatocytes (Figure 5). 

The sinusoids appeared as optically translucent slits, without infiltration. The cytoplasm of the hepatocytes was finely granular, with rounded nuclei and visible nucleoli. Individual dystrophically altered hepatocytes were observed in certain fields of view, displaying moderately hypochromatic cytoplasm and nuclei.

#### 2.2.3. Anti-Inflammatory Activity

The study of the anti-inflammatory activity of the *A. reptans* extracts was conducted using a model of acute aseptic inflammation—carrageenan-induced rat paw edema. The data on the effect of *A. reptans* herb extracts and comparison drugs on the development of rat paw edema, as well as their anti-exudative activity, are presented in Table 5.

The anti-exudative activity of the extracts AR1, AR3, and quercetin manifested itself gradually and reached its maximum within 4 h from the beginning of the experiment, which indicates a moderate effect of these substances on the inhibition of all inflammatory mediators, but mostly on prostaglandins. The activity of the extract AR2 increases within 2 h during the period of release of early inflammatory mediators (kinin and histamine), which confirms the presence of the phenolic compounds we have identified that exhibit antihistamine and polyoxygenase activity. After 2 h, the extract AR2 exceeded the anti-exudative effect of the reference drug quercetin by more than 1.2 times and lasted for 2 h. The effect of diclofenac sodium is manifested immediately, significantly increases after 2 h, and reaches a maximum within 4 h from the beginning of the experiment, which indicates a significant inhibition of all groups of inflammatory mediators. In the control group of animals that did not receive treatment, edema increased within 4 h.

#### 2.2.4. Hemostatic and Wound Healing Activity

The study results on the duration of bleeding (M ± m, seconds) in guinea pigs from a cut wound with the local application of the extracts and the “Rotokan” preparation are presented in Table 6.

The results of the study on the dynamics of the wound healing process with the application of *A. reptans* extracts are presented in Table 7.

*A. reptans* extracts show a local hemostatic effect and improve the wound healing process. AR1 extract was the most promising in reducing bleeding time, while AR2 was the best in the wound healing process.

#### 2.2.5. Antimicrobial and Antifungal Activity

**Figure 6:**
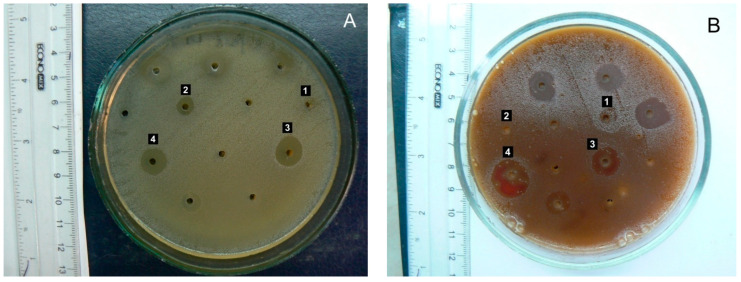
Antimicrobial activity of *Ajuga reptans* L. herb extracts prepared using AR1 (extraction agent: purified water) (1), AR2 (extraction agent: 50% ethanol) (2), AR3 (extraction agent: 70% ethanol) (3), and gentamicin (4) against cultures of *Enterococcus faecalis* (**A**) and β-hemolytic *Streptococcus* group G (**B**).

Bactericidal and bacteriostatic activity of the *A. reptans* herb extracts against various microbial cultures were established. The bactericidal effect was indicated by forming distinct zones of complete inhibition of test culture growth around the wells containing the extracts. In contrast, the bacteriostatic action of the extracts was characterized by zones of partial inhibition of microorganism growth, which lacked clear edges, and atypical colonies formed within these zones (Figure 6 and Table 8).

*A. reptans* extracts demonstrated higher activity against Gram-positive microorganisms, with streptococci being particularly sensitive.

## 3. Discussion

Among the phenolic compounds in the *A. reptans* extracts (Table 1), eight flavonoids were identified: rutin, quercetin, quercetin-3-glucoside, luteolin, apigenin, neohesperidin, naringin, and naringenin. The flavonoids mentioned in Table 1 include four aglycones and four glycosides, but quantitatively, there are more glycosides than aglycones, which are responsible for biological activity [31].

Additionally, four tannin metabolites were identified: pyrocatechin, epicatechin, epicatechin gallate, and gallocatechin. Five hydroxycinnamic acids were also identified: caffeic, *p*-coumaric, sinapic, *trans*-cinnamic, and *trans*-ferulic acids. Three phenol carboxylic acids were found: gallic, benzoic, and syringic acids. Quinic acid was also identified in the extracts. The predominant hydroxycinnamic acids in *A. reptans* herb extracts were *p*-coumaric, caffeic, and sinapic acids; among the flavonoids, rutin, quercetin, and neohesperidin were predominant, while among the tannin metabolites, epicatechin gallate and gallocatechin were the most significant. It was reported that in *A. reptans,* raw materials from Turkey, caffeic and chlorogenic acids predominated among hydroxycinnamic acids and luteolin derivatives—among flavonoids [19]. In the other research, isoquercitrin and ferulic acids were dominant phenolic compounds [6]. Thus, Ukrainian raw materials have a similar qualitative composition but differ in dominant substances.

As shown in Table 2, 10 volatile compounds were identified and quantified in the *A. reptans* herb extracts, including one ketone, two alcohols, and one terpenoid compound. The alcohols and their derivatives included 3-heptanol and α-terpineol; the ketone identified was 2-heptanone. Among sesquiterpenes and their derivatives were α-bergamotene and hexahydrofarnesyl acetone; among aliphatic sesquiterpenes was farnesene; and among terpenoids was α-linalool. The extracts also contained acyclic triterpenes such as squalene and esters like methyl linoleate and linoleic acid. Other research has shown that teupolioside, martinoside, verbascoside, and isoverbascoside are the major chemical constituents among phenylproponoids, and teupolioside is the chemical marker of the *A. reptans* extract, and major monoterpenoids include three iridoid glycosides: harpagide, 8-*O*-acetylharpagide and reptoside [32]. Thus, our results extend the data on the chemical composition of the volatile fraction.

The qualitative composition and quantitative content of amino acids in the extracts of the *A. reptans* herb were studied for the first time. The 17 identified amino acids included ten monoaminomonocarboxylic acids: alanine, valine, glycine, isoleucine, leucine, methionine, serine, threonine, phenylalanine, and cysteine; two monoaminodicarboxylic acids: aspartic and glutamic acids; two diaminomonocarboxylic acids: arginine and lysine; and two heterocyclic amino acids: histidine and tryptophan. According to experimental research data (Table 3), glutamic acid, aspartic acid, arginine, leucine, serine, valine, and glycine predominated in *A. reptans* herb extracts.

The soft extracts of the *A. reptans* herb are classified as practically non-toxic (toxicity class V) when administered intragastrically (LD_50_ > 5000 mg/kg) according to the classification by K.K. Sidorov [29].

The simultaneous administration of the extracts and the hepatotoxic agent led to a reduction (Table 4) in the content of TBA-reactive substances by 1.58, 1.56, and 1.55 times, respectively, and a decrease in ALT activity by 1.52, 1.32, and 1.25 times in the blood serum of the experimental animals and by 1.88, 2.11, and 1.87 times, respectively, in the liver homogenate compared to the control group of animals. The results obtained from the conducted studies indicate that the *A. reptans* herb soft extracts exhibit a pronounced hepatoprotective activity in cases of acute toxic liver damage by suppressing peroxide destructive processes and reducing the development of cytolysis syndrome, and they are practically not inferior to the hepatoprotective action of the comparison drug “Silibor”. The hepatoprotective activity of *A. reptans* herb extracts has been studied for the first time.

The study results (Table 5) show that the anti-exudative activity of AR1, AR3 extracts, and quercetin gradually manifested and reached its maximum within 4 h from the start of the experiment, indicating a moderate effect of these substances. The anti-inflammatory effect is minor and has no pharmacological significance, as a level of pharmacological activity of at least 20% is considered significant for the experimental study of anti-inflammatory agents [33]. The activity of the AR2 extract increased within 2 h during the release period of early inflammation mediators (kinin and histamine), confirming the presence of identified polyphenolic compounds with antihistaminic and polyoxygenase activity. The results indicate that, after 2 h, the AR2 extract exceeded the anti-exudative effect of the reference drug quercetin by more than 1.2 times and maintained this effect for 2 h. The effect of diclofenac sodium manifested immediately, increased significantly within 2 h, and reached its maximum 4 h after the start of the experiment, indicating significant inhibition of all groups of inflammation mediators. In the control group of untreated animals, edema increased within 4 h. The study of the anti-inflammatory activity of the tested extracts established that the AR2 extract at a dose of 50 mg/kg exceeded the anti-exudative effect of the reference drug quercetin (anti-exudative effect—20.52%) after 2 h. Previously, in vivo and in vitro studies have shown that the representatives of the *Ajuga* L. genus inhibit inflammatory responses by suppressing inflammatory factors (cyclooxygenase-1 (COX-1), cyclooxygenase-2 (COX-2), inducible nitric oxide synthase (iNOS), nitric oxide (NO), interleukin 8 (IL-8), interleukin 6 (IL-6), and tumor necrosis factor-α (TNF-α) [4,34]. Such data are available for some *Ajuga* species but are lacking them for *A. reptans.* The best anti-oxidative and anti-inflammatory activity was observed for *the A. reptans* 100 mg dw/mL extract when compared with diclofenac [6], but in our research the effective therapeutic dose was 50 mg/kg.

The obtained results (Table 6) indicate that the local application of *A. reptans* extracts (using a gauze pad soaked in the tested extracts directly on the wound surface immediately after the wound was made), significantly decreased bleeding time compared to the control group of animals. The most significant reduction in bleeding was caused by applying the AR1 extract, reducing bleeding time by 40.59%, while the hemostatic activity of the “Rotokan” preparation reduced it by 52.61% compared to the control group. Our conducted studies indicate that *A. reptans* extracts have a local hemostatic effect. The study’s results on the dynamics of the wound healing process with the application of *A. reptans* extracts (Table 7) show that from the 9th day of the local application of the AR2 extract and the comparison drug “Rotokan”, complete wound healing occurred. There was no scientific confirmation of the hemostatic and wound healing activity of *A. reptans* extracts, although, in folk medicine, it is commonly recommended in these cases [4].

The conducted studies established that *A. reptans* herb extracts can inhibit the growth of microorganisms to varying degrees, depending on the ethanol concentration used as an extraction agent.

The AR3 herb extract (extraction agent: 70% ethanol) caused significant inhibition of the growth of pathogenic β-hemolytic *Streptococcus* of groups A and G—causative agents of tonsillitis and angina (Figure 3) as well as β-hemolytic *Streptococcus* group B—a causative agent of inflammatory processes in the female external genital organs. Conditionally pathogenic α-hemolytic *Streptococcus* of the oral microflora, *St. oralis*, *St. sanguinis*, and *St. gordonii* (which can cause purulent-inflammatory processes of the oral mucosa and periodontal tissues in dental practice) also showed high sensitivity to the AR3 extract. The growth of *S. sanguinis* and *St. oralis* was also notably inhibited by the AR2 extract. Regarding the primary causative agent of bacterial respiratory infections (bronchitis, otitis, sinusitis), *Pneumococcus St. pneumonia*, the AR2 and AR3 extracts exhibited a bacteriostatic effect. Therefore, it can be suggested that *A. reptans* herb extracts have potential use in treating streptococcal infections in dental, pediatric, and ENT practices.

*Enterococcus faecalis*, a common causative agent of urological and wound infections, demonstrated quite high sensitivity to AR3. This result deserves special attention due to enterococcus’s high natural resistance to most antibiotic groups.

The most common causative agents of purulent-inflammatory processes, *Staphylococci*, were significantly less sensitive to *A. reptans* herb extracts than *Streptococci*. *S. aureus* and *S. haemolyticus* exhibited weak sensitivity to all tested preparations.

Gram-negative bacteria generally showed significantly lower sensitivity to the BAS of *A. reptans* extracts. Normal *E. coli* was found to be completely insensitive to them. However, there was a tendency for AR3 to inhibit the growth of *E. fergusonii*, which has reduced enzymatic activity, as well as *Citrobacter* and representatives of the putrefactive intestinal microflora *Providencia* and *Morganella*. Therefore, the inclusion of the *A. reptans* herb as an auxiliary component can be considered when developing herbal mixtures and complex herbal remedies for treating mild forms of intestinal dysbiosis [35,36].

The antibacterial effects of the essential oil isolated from the aerial part of *A. pseudoiva* have been tested before against Gram-positive and Gram-negative bacteria in vitro, including *B. subtilis*, *B. cereus*, *S. aureus*, *E. coli*, *E. faecalis*, *K. pneumonia*, *P. aeruginosa*, *S. typhimurium*, *L. monocytogenes*, and *E. faecium* [4,37]. The ethanol and methanol extracts from the aerial parts of *A. laxmannii* have been shown to exhibit antibacterial activity against *P. aeruginosa*, *L. monocytogenes*, *E. coli*, and *S. typhimurium* [6]. Thus, the results of the antibacterial effects of *A. reptans* herb extracts provide new knowledge.

The *A. reptans* herb extracts showed weak fungistatic activity against most types of *Candida* yeast-like fungi. Previously, it was also shown that *A. reptans* extracts are active against *Canada* spp. and *E. coli* [19,32].

During the conducted studies, an important pattern was established: the antimicrobial activity of *A. reptans* herb extracts increases proportionally with the increase in ethanol concentration in the extracting solution. This may indicate the predominantly hydrophobic properties of BAS provide the antimicrobial activity of the extracts.

The study has some limitations. The sample of *A. reptans* we used was collected only from one growing locality. We presented the phytochemical characterization of the sample, but the concentration of bioactive substances is less or more different due to environmental factors and possible chemotypes. In future studies, it may be more adequate to analyze a mixture of herbal samples collected from several localities. Also, the other extraction solvents excluding water, 50% and 70% ethanol, and methods affect the phytochemical composition and pharmacological activity of the extracts to some extent. Biological mechanisms have not been the focus of the study; thus, many additional studies with a specific focus are needed in the future. The authors hope that our study will provide a significant basis for *A. reptans* investigations in the future, using additional plant material, methods, and activities.

## 4. Materials and Methods

### 4.1. Chemicals

Deionized water was produced using the Millipore Simplicty UV station (Merck Millipore, Burlington, MA, USA). Ethanol, methanol, acetonitrile, formic acid, and carbon tetrachloride originated from VWR (Radnor, PA, USA). Standards of malic, gallic, and chlorogenic acids, rutin (Sigma-Aldrich Products, Burlington, MA, USA), and standard solutions of amino acids (TU 6-09-3147-83) were used for analysis. λ-carrageenan produced by Fluka (Buchs, Switzerland) was used. For measuring ALT and AST, a set of reagents from the “SIMKO Ltd.” company (Lviv, Ukraine) and TBA-reactive substances biochemical kits from the Felicit-Diagnostics (Dnipro, Ukraine) were used. Bacterial cultures were identified based on the biochemical microtests “STAPHYtest 16”, “ENTEROtest 24”, and “NEFERMENTtest 24” (Lachema, Czech Republic). Cultures of yeast-like fungi were identified based on 40 biochemical tests using the VITEK 2 system with VITEK 2 YST ID cards (bioMérieux, Craponne, France). Vikasol solution for injection, 10 mg/mL (Darnitsa, Kyiv, Ukraine); Silibor film-coated tablets, 35 mg, of the *Silybum marianum* extract (Pharmaceutical company «Zdorovye», Kharkiv, Ukraine); Rotokan liquid extract, 55 mL (Lubnyfarm, Lubny, Ukraine), which is a mixture of liquid extracts of *flores Chamomile*, *flores Calendulae*, and *herba Millefolii* (2:1:1); diclofenac sodium solution for injection, 25 mg/mL (Darnitsa, Kyiv, Ukraine) were used as reference medicines in the pharmacological research.

### 4.2. Plant Raw Materials

The herb of *A. reptans* L. was collected during the flowering period in 2021 in the vicinity of the village of Huta, Bohorodchany District, Ivano-Frankivsk Region (48°39′4″ N, 24°12′55″ E 48.651111°, 24.215278°). The herb was collected in the phase of mass flowering and consists of pieces of stems, leaves, and flowers. The harvesting of raw materials was carried out in accordance with the collection guidelines, taking into account the specifics of harvesting and ensuring careful handling of the flora. Drying of the medicinal raw material was performed in the shade outdoors, avoiding direct sunlight, and it was stored in paper bags. A total of 2 kg of the dry raw material was obtained. According to the botanical catalog, the plant’s identity was confirmed with the consulting assistance of Professor A.R. Grytsyk from Ivano-Frankivsk National Medical University (IFNMU) [38]. Voucher specimens No. 551–553 were deposited at the Department of Pharmaceutical Management, Drug Technology, and Pharmacognosy at Ivano-Frankivsk National Medical University.

### 4.3. Extracts Preparation

To prepare the extracts, the herb of *A. reptans* (with a mass loss on drying of 10–12%) was ground to particles of 0.5–2.5 mm. The studied raw material was extracted with purified water [25], 50% and 70% ethanol solutions, for 30 min in a water bath with a reflux condenser. The ratio of raw material to extractant was 1:15 for purified water and 1:10 for 50% and 70% ethanol. The extraction was performed three times, and the extracts were combined. The extracts were purified by allowing them to settle at a temperature not exceeding 10 °C for 48 h to remove ballast substances. Subsequently, the settled extracts were filtered. The filtrates were evaporated using a rotary evaporator IR-1M2 to achieve a viscous consistency in two stages. Initially, the entire volume of extracts was evaporated to remove all ethanol. This process was conducted at a temperature of 60–70 °C under reduced pressure of 0.55–0.6 MPa. The degree of evaporation was visually monitored by the volume of alcohol condensate and by measuring alcohol concentration using an alcohol meter in condensate samples. Evaporation continued until no alcohol was detected in the samples. After removing the alcohol from the extracts, evaporation continued at a temperature of 70–80 °C under reduced pressure of 0.6–0.65 MPa. The degree of evaporation was again monitored visually by the volume of water condensate. The finished extracts were poured into wide-neck jars with tight-fitting lids and labeled.

### 4.4. Phytochemical Research

#### 4.4.1. HPLC Methods

Previously, the high-performance thin-layer chromatography (HPTLC) method was used to identify phenolic compounds in the extracts. Chromatographic plates of MERCK Silica gel F254 and a solvent system consisting of anhydrous formic acid, water, and ethyl acetate (10:10:80) were used for chromatography. A solution of 10 g/L diphenylboric acid aminoethyl ester in methanol and 50 g/L macrogol 400 P in methanol was used to develop the chromatograms. The results were evaluated by comparing the R_f_ values of the zones on the chromatogram of the test solution with those of the reference solution. The following reference substances were used for preparing the comparison solution: rutin, chlorogenic acid, hyperoside, apigenin-7-glucoside, ferulic acid, luteolin, apigenin, luteolin-7-glucoside, caffeic acid, and quercetin [39,40].

Flavonoids were studied using high-performance liquid chromatography (HPLC) on an Agilent Technologies 1200 liquid chromatograph. Acetonitrile (A) and 0.1% formic acid solution in water (B) were used as the mobile phase. Elution was carried out in gradient mode: 0 min—A (30%): B (70%); 20 min—A (70%): B (30%); 22 min—A (100%): B (0%); 30 min—A (100%): B (0%). Separation was performed on a Zorbax SB-C18 chromatographic column (3.5 µm, 150 × 4.6 mm) (Agilent Technologies, Santa Clara, CA, USA) with a flow rate of 0.25 mL/min. The sample injection volume was 100 µL, and the column temperature was maintained at 25 °C. Detection was conducted using a diode-array detector with signal registration at 280 and 365 nm and spectra recorded in the range of 210–270 nm [41,42].

The study of hydroxycinnamic acids was carried out using HPLC on an Agilent Technologies 1200 liquid chromatograph. Methanol (A) and 0.1% formic acid solution in water (B) were used as the mobile phase. Elution was conducted in a gradient mode: 0 min—A (25%): B (75%); 25 min—A (75%): B (25%); 27 min—A (100%): B (0%); 35 min—A (100%): B (0%). Separation was performed on a Zorbax SB-Aq chromatographic column (4.6 mm × 150 mm, 3.5 µm) (Agilent Technologies, USA) with a flow rate of 0.5 mL/min, a column temperature of 30 °C, and an injection volume of 4 µL. Detection was conducted using a diode-array detector with signal registration at 250 and 275 nm and absorption spectra fixed in the range of 210–270 nm [43,44].

The study of tannin metabolites was carried out on an Agilent Technologies 1200 liquid chromatograph. Methanol (A) and 0.1% formic acid solution in water (B) were used as the mobile phase. Elution was conducted in a gradient mode: 0 min—A (20%):B (80%); 25 min—A (75%):B (25%); 27 min—A (100%):B (0%); 35 min—A (100%):B (0%). Separation was performed on a Zorbax SB-C18 chromatographic column (3.5 µm, 150 × 4.6 mm) (Agilent Technologies, USA) with a flow rate of 0.25 mL/min, a thermostat temperature of 35 °C, and an injection volume of 4 µL. Detection was conducted using a diode-array detector with signal registration at 250 and 275 nm and absorption spectra fixed in the range of 210–270 nm [43].

Identification and quantitative analysis of substances were conducted by comparison with reference standards of quinic, gallic, benzoic, syringic, caffeic, *p*-coumaric, *trans*-ferulic, sinapic, and *trans*-cinnamic acids; rutin; quercetin-3-glucoside; naringin; neohesperidin; quercetin; luteolin; apigenin; naringenin; pyrocatechin; epicatechin; epicatechin gallate; and gallocatechin (Sigma-Aldrich Products, Burlington, MA, USA). For statistical processing of data, measurements were repeated three times.

The identification and determination of the content of amino acids in *A. reptans* herb extracts was carried out based on the SE “Ivano-Frankivsk Scientific and Production Center for Standardization, Metrology and Certification” (accreditation certificate No. 2H098 dated 20 June 2014) [20,21]. Analysis was conducted using an amino acid analyzer AAA T-339 M. (Mikrotekhnika, Prague, Czech Republic) compared with the concentration of standard amino acid hydrolysates according to DSTU ISO 13903:2005. Quantification was performed using standard solutions of amino acids (TU 6-09-3147-83) [45].

#### 4.4.2. GC-MS Methods

Volatile compounds were researched using gas chromatography-mass spectrometry (GC-MS). The qualitative composition and content (mg/kg) of volatile compounds were determined using gas chromatography-mass spectrometry (GC-MS) on an Agilent Technologies 6890 chromatograph with a mass spectrometric detector 5973. A capillary HP-5ms chromatographic column with an internal diameter of 0.25 mm (film thickness 0.25 µm) and a length of 30 m was used. The carrier gas, helium, had a flow rate of 1.0 mL/min. The sample injection heater was maintained at a temperature of 250 °C. The column’s thermostat temperature was programmed to increase from 60 °C to 320 °C at a rate of 7 °C/min. Components were identified based on the general patterns of molecular fragmentation of organic compounds under electron impact and by comparing the obtained spectra with the data from the NIST05 and WILEY 2007 mass spectra libraries, which contain over 470,000 spectra. The match of the mass spectra with the databases was not less than 90%. The AMDIS and NIST identification software programs were used for data analysis [28]. The analysis of the volatile fraction was performed using the European Pharmacopeia method, which requires only one sample to be analyzed [25]. The error of the GC apparatus itself is less than 1%.

#### 4.4.3. Spectrophotometric and Titrimetric Methods

The quantitative determination of vitamin K content recalculated to Vikasol equivalents in the studied samples of *A. reptans* herb extracts was carried out using the spectrophotometric method [46].

The content of free organic acids, recalculated as malic acid equivalents, in the extracts was determined by titration according to the pharmacopeial method [24,47].

The quantitative determination of ascorbic acid was carried out using the titrimetric method, following the pharmacopeial procedure [48,49].

The quantitative determination of tannins and total phenolic content, expressed as pyrogallol equivalents, was performed using the spectrophotometric method by the pharmacopeial method DFU 2.0 [24,25].

The quantitative determination of the total flavonoid content, expressed as rutin equivalents, was performed using the spectrophotometric method according to the pharmacopeial method DFU 2.0 [24,50].

The quantitative determination of hydroxycinnamic acids, expressed as chlorogenic acid equivalents, in the extracts was conducted using the spectrophotometric method at a wavelength of 325 nm [51,52].

For statistical processing of data, all the measurements were repeated three times. The detailed description of the assays is presented in Supplementary Material S1.

### 4.5. Pharmacological Research

The pharmacological and toxicological properties of *A. reptans* herb extracts were studied according to the “General Ethical Principles of Experiments on Animals” (Ukraine, 2001), which comply with the provisions of the “European Convention for the Protection of Vertebrate Animals Used for Experimental and Other Scientific Purposes” (Strasbourg, 1986) [52,53,54]. Laboratory animals were kept according to the current “Sanitary Rules for the Arrangement, Equipment, and Maintenance of Experimental Biological Clinics (Vivaria)” at a temperature of 18–20 °C and relative humidity of 50–55%. Feeding was conducted with a complete diet according to a standard scheme. Before the experiment began, the animals underwent a 7-day adaptation period during which their clinical condition was carefully observed daily. Throughout the study, observations were made regarding the clinical condition and behavior of the experimental animals. After the end of the experiment, the animals were euthanized via decapitation under ether anesthesia. The research was approved by the Bioethics Commission of the Ivano-Frankivsk National Medical University (protocol №121/21 from 13 May 2021).

#### 4.5.1. Acute Toxicity

The acute toxicity of *A. reptans* herb extracts was studied with the consulting assistance of Professor A.O. Klymenko from the Department of Biological and Medical Chemistry named after Academician H.O. Babenko, Professor O.G. Popadynets from the Department of Human Anatomy, and Professor Ya.S. Hudyvok from the Department of Pharmacology at IFNMU.

Biochemical parameters were studied at the Bioelementology Center of the Ivano-Frankivsk National Medical University (accreditation certificate No. 037/14 dated 29 April 2014). Laboratory mice weighing 19–23 g were used in the experiment. To determine the parameters of acute toxicity of *A. reptans* herb extracts, the animals were divided into four groups (6 animals in each group):

- Group I—intact animals (purified water);
- Group II—AR1 extract (extraction agent—purified water);
- Group III—AR2 extract (extraction agent—50% ethanol);
- Group IV—AR3 extract (extraction agent—70% ethanol);

The solutions of the substances were administered intragastrically using a gavage, ensuring that the solution volume did not exceed 0.5 mL. The dose was calculated in mg of active substance per 1 kg of body weight. Observations were conducted over 14 days. After the experiment, the animals were euthanized via decapitation under ether anesthesia. Internal organs were examined, and blood samples were collected for biochemical analysis [29].

#### 4.5.2. Hepatoprotective Activity

The study of the hepatoprotective activity of *A. reptans* L. herb extracts—AR1 (extraction agent: purified water), AR2 (extraction agent: 50% ethanol), and AR3 (extraction agent: 70% ethanol)—was conducted using a model of acute carbon tetrachloride (CCl_4_) hepatitis [30,55]. The domestically produced hepatoprotective agent “Silibor” was used as a comparison drug.

The experiments were conducted on male white rats weighing 0.18–0.22 kg, divided into six groups (6 animals in each group). Animals in Groups I–III were administered *A. reptans* L. herb extracts (extraction agents: purified water, 50% ethanol, and 70% ethanol, respectively). The animals in Group IV received the comparison drug “Silibor” at a dose of 25 mg/kg. The extracts and the comparison drug were administered intragastrically via oral gavage as a water solution or suspension, which was prepared immediately before consumption. Group V was the control group that received no treatment, just 50% oily carbon tetrachloride solution, and Group VI consisted of intact animals. Liver pathology was induced by a single subcutaneous injection of a 50% oily carbon tetrachloride solution at a dose of 0.8 mL per 0.1 kg of body weight to the animals in the first to fifth groups over two days with a 24 h interval. The studied extracts and the comparison drug were administered one hour before and two hours after the introduction of the hepatotropic agent.

The choice of carbon tetrachloride as the intoxicant was based on its high selective hepatotoxicity. Poisoning of experimental animals with this xenobiotic leads to morphological and biochemical changes that closely resemble acute liver damage of various etiologies in humans [56].

The animals were euthanized by decapitation on the third day after the first injection of carbon tetrachloride, after which blood samples were collected, and the liver was isolated. The effectiveness of the investigated extracts was assessed based on biochemical and functional parameters of the liver and blood serum, which were determined 24 h after the last injection of carbon tetrachloride.

One of the criteria for evaluating the hepatoprotective effect of the studied extracts was the survival rate of the animals. The mortality rate in the control group was 16.7%, while all animals in the other groups survived until the end of the experiment. The intensity of oxidative destructive changes in the animals’ bodies was assessed by measuring the content of thiobarbituric acid (TBA) reactive substances in blood serum and liver homogenate. The activity of alanine aminotransferase (ALT), which is a hepatospecific marker of cytolysis, was also determined in blood serum. The ALT activity was measured using Reitman and Frankel’s unified dinitrophenylhydrazine method with a standard set of reagents from the “SIMKO Ltd.” company [30].

The level of lipid peroxidation products, specifically TBA-reactive substances, was assessed by their reaction with 2-thiobarbituric acid using a spectrophotometric method according to the technique developed by E.N. Korobeynikova, using biochemical kits from a domestic manufacturer (“Felicit-Diagnostics”, Ukraine) [57].

#### 4.5.3. Anti-Inflammatory Activity

The study of the anti-inflammatory activity of *A. reptans* extracts was conducted with the consultative assistance of Dr. H.M. Ersteniuk, Doctor of Biological Sciences, Professor of the Department of Biological and Medical Chemistry named after Academician H.O. Babenko.

The experiment was conducted on non-linear white rats of both sexes weighing 180–220 g (*n* = 48), which were standardized according to physiological and biochemical parameters. The animals were kept under vivarium conditions in accordance with sanitary and hygienic norms.

To assess the impact of *A. reptans* extracts on the progression of the exudative phase of the inflammatory process, a model of acute aseptic inflammation—carrageenan-induced rat paw edema—was employed. λ-carrageenan produced by Fluka (Switzerland) was used. This model is characterized by the cyclooxygenase pathway of inflammation as described in the methodology by Trynus F.P. et al. [58]. In this model, 0.1 mL of a 1% carrageenan solution was injected subplantarly into the paw pad of a rat under aseptic conditions to induce inflammation. Carrageenan-induced edema inhibits the synthesis of serotonin, kinins, histamine, and prostaglandins. Serotonin and histamine are inhibited within 0.5–1.5 h; kinins between 1.5 and 2.5 h; and prostaglandins from 2.5 to 5.5 h.

Using a mechanical oncometer developed by A.S. Zakharevskyi, changes in the rat’s paw volume were measured at the start of the study and dynamically 1, 2, and 4 h after introducing the phlogogenic agent. The changes in animals receiving the test preparations were compared with those in the control group. Results were calculated and expressed as percentages [30].

For comparison, under similar conditions, the anti-exudative effect of quercetin, a plant-derived preparation with proven anti-exudative activity, and diclofenac sodium, a standard nonsteroidal anti-inflammatory drug, was studied [59].

The experimental animals were divided into six groups, with six animals in each group. The animals in the first to third groups were administered extracts AR1 (extraction agent: purified water), AR2 (extraction agent: 50% ethanol), and AR3 (extraction agent: 70% ethanol), respectively, at a single dose of 100 mg/kg, as a water solution or a water suspension. The fourth group received quercetin at a dose of 50 mg/kg of body weight, and the fifth group received diclofenac sodium at a dose of 8 mg/kg of body weight. The sixth group served as the control group. Forty minutes before the injection of the phlogogenic agent, the animals were administered intragastrically the single dose of the test extracts and comparison drugs according to the group assignment. Control animals received an equivalent volume of physiological solution.

The anti-exudative activity of the substances tested was determined as a percentage of the degree of reduction in edema in the animals that received the test preparations compared to the control group [29,45].

#### 4.5.4. Research on Hemostatic and Wound-Healing Effects

The study of the wound-healing and hemostatic properties of *A. reptans* herb extracts was conducted with the consultative assistance of Professor O.G. Popadynets from the Department of Human Anatomy and Professor Ya.S. Hudyvok from the Department of Pharmacology at IFNMU.

The wound-healing activity of *A. reptans* herb extracts was investigated on clinically healthy guinea pigs over a 16-day period. The tested extracts were applied twice a day, starting from the first day until complete healing. Dynamic observation was conducted on the 3rd, 5th, 7th, 9th, 14th, and 16th days to evaluate the inflammatory response, conduct planimetry, and determine the rate of wound healing [60]. The preparation of “Rotokan”, a combined herbal medicine containing tinctures of chamomile flowers, calendula flowers, and yarrow herb, was used as a comparison.

A linear incision wound (wound model) was made by cutting through all skin layers and the muscle layer on the lateral surface of the abdomen using a scalpel. The operational area was pre-shaved, treated with a 5% iodine solution, and anesthetized using barbamil anesthesia (0.8 mL per 100 mg of a 1% aqueous barbamil solution). The size of the model wound was 1.5 × 0.3 cm. The duration of bleeding was determined by measuring the time (using Duke’s method) from the moment of tissue incision until spontaneous hemostasis [29].

#### 4.5.5. Research on Antimicrobial Activity and Microbiological Purity

The study of the potential antimicrobial activity of *A. reptans* herb extracts against the growth of pure cultures of Gram-positive bacteria and yeast was conducted at the Department of Microbiology, Virology, and Immunology at IFNMU, with the consultative assistance of the head of the department, Doctor of Medical Sciences, Professor R.V. Kutsyk.

The objective of the study was to assess the antimicrobial activity of the aqueous extract AR1 and the aqueous-ethanol extracts AR2 and AR3. The antimicrobial activity of the extracts was evaluated using both clinical isolates of antibiotic-sensitive and antibiotic-resistant microorganisms and standard strains. Bacterial cultures were identified based on the biochemical microtests “STAPHYtest 16”, “ENTEROtest 24”, and “NEFERMENTtest 24” (Lachema, Czech Republic), as well as by considering a set of morphological and cultural characteristics according to the recommendations of the 9th edition of “Bergey’s Manual of Determinative Bacteriology” [61]. Cultures of yeast-like fungi were identified based on 40 biochemical tests using the VITEK 2 system with VITEK 2 YST ID cards (bioMérieux, France).

The antimicrobial effect of the plant extracts was examined using the agar diffusion micromethod. This method is highly sensitive and allows reliable differentiation between active and inactive extracts [62]. Petri dishes placed on a horizontal, flat surface were filled with 30 mL of agar. After the medium solidified, wells with a diameter of 4.0 mm were created using a special punch with equal edges. The agar was uniformly seeded with a suspension of the test culture (concentration 1 × 10^7^ CFU/mL). To the experimental wells, 20 μL of 1% of the plant extract water suspensions were added. As during the preparation of the soft extracts, the extractants (50% and 70% ethanol) were evaporated and their 1% water solution or suspensions were used, it makes no sense to compare the extracts’ activity with 50% or 70% ethanol. After 24 h of cultivation, the diameters of the inhibition zones for bacterial test cultures were determined. Fungistatic activity was registered after 2 days, and fungicidal activity was registered after 4 days of cultivation. Gentamicin and fluconazole were used as reference drugs. Digital images of the cultures on the plates were obtained and processed using the computer program Image Tool 2.0 (UTHSCSA ImageTool 2.0, The University of Texas Health Science Center in San Antonio, 1995–1996).

The microbiological purity of the extracts was determined according to the requirements of the State Pharmacopeia of Ukraine 5.1.4 N 2.6.13 N. Category 3 B [24].

### 4.6. Statistical Analysis

Statistical data of random variables with an n-dimensional normal distribution are presented by their correlation matrices, which are calculated from the original matrices. The pharmacological results were calculated using variational statistics. The average mean and its standard deviation, as the reliability of the compared values, were calculated using the Student, Mann–Whitney, and Wilcoxon criteria with a probability level of ≤0.05 on a computer using Statistica 6.0 and Word Excel 2013 programs [24,63]. For statistical processing of data, measurements were repeated at least three times.

## 5. Conclusions

Phytochemical and pharmacological investigations have demonstrated that *A. reptans* L. herb extracts are promising agents with hepatoprotective, antimicrobial, hemostatic, and wound-healing activity to be implemented in medical and pharmaceutical practice. Comprehensive analyses of the soft aqueous and aqueous-alcohol extracts of the *A. reptans* herb identified 16 amino acids, 20 phenolic compounds, and 10 volatile substances. The quantitative content of carboxylic acids, amino acids, ascorbic acid, vitamin K, phytosteroids, and phenolic compounds (flavonoids, hydroxycinnamic acids, and tannins) was established. The soft extract of the *A. reptans* herb (extractant 50% ethanol solution) demonstrated the most pronounced hepatoprotective, anti-inflammatory, and wound-healing effects. In contrast, the soft aqueous extract exhibited superior hemostatic activity. Additionally, *A. reptans* herb extracts effectively inhibited the growth of microorganisms and demonstrated higher activity against Gram-positive microorganisms. The conducted studies showed the promising potential of the obtained extracts, but further preclinical and clinical studies need to be carried out before suggesting applications in clinical medicine.

## Figures and Tables

**Table 1:** The content of biologically active substances in the extracts of the *Ajuga reptans* L. herb.

№	Name of Identified Compound	Quantitative Content, mg/100 g		
		AR1	AR2	AR3
Carboxylic Acid				
1	Quinic acid	146.31 ± 4.12	158.87 ± 9.43	149.14 ± 11.76
Phenolic Acids				
2	Gallic acid	48.03 ± 2.33	68.44 ± 4.54	83.23 ± 6.52
3	Benzoic acid	102.22 ± 7.09	168.59 ± 11.29	184.82 ± 9.07
4	Syringic acid	27.28 ± 1.62	33.22 ± 1.58	41.96 ± 2.01
Hydroxycinnamic Acids				
5	Caffeic acid	326.48 ± 12.17	343.60 ± 31.99	311.62 ± 23.07
6	*p*-Coumaric acid	420.62 ± 23.22	432.63 ± 28.32	376.96 ± 31.44
7	*trans*-Ferulic acid	79.96 ± 5.13	87.22 ± 5.65	64.74 ± 2.12
8	Sinapic acid	261.56 ± 21.12	284.43 ± 21.45	398.23 ± 27.87
9	*trans*-Cinnamic acid	113.87 ± 5.08	126.27 ± 7.67	107.34 ± 12.61
Flavonoids				
10	Rutin	2384.71 ± 207.87	3465.43 ± 167.34	3256.74 ± 267.21
11	Quercetin-3-glucoside	166.52 ± 6.14	173.25 ± 23.45	208.45 ± 26.32
12	Naringin	146.97 ± 5.18	156.43 ± 9.23	187.97 ± 20.02
13	Neohesperidin	330.67 ± 12.16	374.28 ± 27.65	401.27 ± 31.89
14	Quercetin	1325.53 ± 115.54	1453.22 ± 117.39	1543.64 ± 107.86
15	Luteolin	312.32 ± 21.62	341.57 ± 25.02	376.54 ± 28.51
16	Apigenin	117.85 ± 7.02	134.67 ± 8.48	153.75 ± 18.84
17	Naringenin	100.51 ± 4.89	112.92 ± 10.12	132.83 ± 10.08
Tannin Metabolites				
18	Pyrocatechin	53.44 ± 2.45	65.21 ± 8.55	77.15 ± 4.45
19	Epicatechin	395.93 ± 30.34	356.78 ± 12.68	401.48 ± 36.33
20	Epicatechin gallate	1295.58 ± 108.32	1129.42 ± 159.56	1209.21 ± 143.67
21	Gallocatechin	1079.72 ± 76.28	985.46 ± 76.09	1067.53 ± 109.78
Group content, %				
Vitamin K_1_ (Vikasol equivalents)		0.73 ± 0.02	0.81 ± 0.04	0.95 ± 0.07
Organic acids (malic acid equivalents)		2.29 ± 0.05	2.03 ± 0.04	1.89 ± 0.04
Ascorbic acid (titrimetry)		0.13 ± 0.01	0.09 ± 0.02	0.08 ± 0.01
Total polyphenols (pyrogallol equivalents)		14.25 ± 0.74	15.11 ± 0.57	15.41 ± 0.82
Total tannins (pyrogallol equivalents)		3.72 ± 0.30	3.08 ± 0.31	2.81 ± 0.21
Total flavonoids (rutin equivalents)		5.37 ± 0.76	6.93 ± 0.48	7.07 ± 0.71
Total hydroxycinnamic acids (chlorogenic acid equivalents)		5.58 ± 0.07	5.91 ± 0.09	5.80 ± 0.03

Notes: AR1—the soft extract from *A. reptans* L. herb (extractant water); AR2—the soft extract from *A. reptans* L. herb (extractant 50% ethanol); AR3—the soft extract from *A. reptans* L. herb (extractant 70% ethanol).

**Table 2:** The content of volatile substances and phytosterols in the extracts of the *Ajuga reptans* L. herb.

Substance Name	Class	Content, mg/kg	
		AR2	AR3
2-Heptanone	Ketones	11.56	13.43
3-Heptanol	Alcohols	4.49	5.78
α-Linalool	Terpenoids	13.30	17.83
α-Terpineol	Monoterpenic alcohols	9.36	10.12
Farnesene	Aliphatic sesquiterpenes	7.90	9.33
Hexahydrofarnesyl acetone	Sesquiterpene derivatives	83.55	96.32
α-Bergamotene	Sesquiterpenes	6.83	9.71
Linoleic acid	Esters	77.19	68.23
Methyl linoleate	Esters	138.97	141.52
Squalene	Acyclic triterpene	15.38	21.83

Notes: AR2—the soft extract from *A. reptans* L. herb (extractant 50% ethanol); AR3—the soft extract from *A. reptans* L. herb (extractant 70% ethanol).

**Table 3:** Amino acid content in the extracts of the *Ajuga reptans* L. herb.

Amino Acid	Chemical Formula	Content of Free Amino Acids, mg/100 g		
		AR1	AR2	AR3
Monoaminomonocarboxylic				
Alanine	C_3_H_7_O_2_N	14.03 ± 0.07	11.83 ± 0.09	10.98 ± 0.08
Valine	C_5_H_11_O_2_N	12.67 ± 0.07	14.67 ± 0.07	16.82 ± 0.04
Glycine	C_2_H_5_O_2_N	15.45 ± 0.15	13.81 ± 0.06	12.89 ± 0.11
Isoleucine	C_6_H_13_O_2_N	11.93 ± 0.06	11.64 ± 0.03	10.81 ± 0.10
Leucine	C_6_H_13_O_2_N	19.73 ± 0.17	18.21 ± 0.02	17.88 ± 0.07
Methionine	C_5_H_11_O_2_NS	9.07 ± 0.06	7.23 ± 0.03	7.82 ± 0.12
Serine	C_3_H_7_O_3_N	19.03 ± 0.14	17.13 ± 0.11	17.86 ± 0.16
Threonine	C_4_H_9_O_3_N	11.04 ± 0.11	10.33 ± 0.08	10.72 ± 0.09
Phenylalanine	C_9_H_11_O_2_N	10.06 ± 0.09	11.81 ± 0.06	13.58 ± 0.13
Cysteine	C_6_H_12_N_2_O_4_S_2_	3.82 ± 0.07	4.11 ± 0.07	4.67 ± 0.07
Total		126.83	120.77	124.03
Monoaminodicarboxylic				
Aspartic acid	C_4_H_7_O_4_N	29.81 ± 0.21	28.93 ± 0.13	26.09 ± 0.19
Glutamic acid	C_5_H_9_O_4_N	49.87 ± 0.18	54.91 ± 0.22	55.83 ± 0.15
Total		79.87	83.84	81.92
Diaminomonocarboxylic				
Arginine	C_6_H_14_O_2_N	33.76 ± 0.17	35.16 ± 0.09	41.82 ± 0.13
Lysine	C_6_H_14_O_2_N	9.53 ± 0.07	8.71 ± 0.07	9.07 ± 0.08
Total		43.29	43.87	50.89
Heterocyclic				
Histidine	C_6_H_9_O_2_N	8.83 ± 0.08	9.64 ± 0.06	9.87 ± 0.04
Tryptophan	C_11_H_12_N_2_O_2_	0.71 ± 0.02	0.93 ± 0.02	0.89 ± 0.04
Total		9.54	10.57	10.76
Overall total content		259.53	259.05	267.60

Notes: AR1—the soft extract from *A. reptans* L. herb (extractant water); AR2—the soft extract from *A. reptans* L. herb (extractant 50% ethanol); AR3—the soft extract from *A. reptans* L. herb (extractant 70% ethanol).

**Table 4:** The effect of *Ajuga reptans* L. extracts on the biochemical parameters of blood serum and liver condition in acute carbon tetrachloride-induced hepatitis.

Biochemical and Hematological Indicators	Research Objects					
	Silibor	AR1	AR2	AR3	Control Animal Group (50% Oil Solution CCl_4_)	Intact Animals
Number of animals	6	6	6	6	6	6
Dose, mg/0.1 kg	2.5	2.5	2.5	2.5	0.8 mL	-
ALT, µmol/h.mL	3.70 ± 0.18 */**	4.01 ± 0.19 */**	4.61 ± 0.12 */**	4.89 ± 0.19 */**	6.12 ± 0.23 *	1.07 ± 0.04
AST, µmol/h.mL	3.25 ± 0.15 */**	2.30 ± 0.10 */**	2.93 ± 0.11 */**	3.83 ± 0.14 */**	4.03 ± 0.15 *	1.13 ± 0.04
TBA-reactive substances, nmol/mL	3.45 ± 0.12 */**	3.42 ± 0.12 */**	3.52 ± 0.17 */**	3.50 ± 0.12 */**	5.47 ± 0.27 *	3.27 ± 0.11
Arginase, µmol/0.1 mL	0.85 ± 0.04 */**	0.61 ± 0.02 */**	0.70 ± 0.03 */**	0.68 ± 0.01 */**	1.21 ± 0.09 *	0.58 ± 0.02
Ceruloplasmin, u.o.	25.5 ± 0.95 */**	25.41 ± 0.98 */**	24.89 ± 0.85 */**	25.01 ± 0.97 */**	19.01 ± 0.78 *	27.00 ± 0.84
Liver Homogenate						
TBA-reactive substances, µmol/g	34.15 ± 1.23 */**	30.35 ± 1.14 */**	34.06 ± 1.12 */**	34.23 ± 1.16 */**	64.31 ± 2.98 *	27.23 ± 0.88

Notes: *—statistically significant difference compared to the data of the intact animal group (*p* ≤ 0.05); **—statistically significant difference compared to the data of the control animal group (*p* ≤ 0.05). AR1—the soft extract from *A. reptans* L. herb (extractant water); AR2—the soft extract from *A. reptans* L. herb (extractant 50% ethanol); AR3—the soft extract from *A. reptans* L. herb (extractant 70% ethanol).

**Table 5:** The effect of *Ajuga reptans* L. extracts on the increase in paw volume and suppression of the inflammatory response in rat limbs.

Group №	Tested Substance	Dose, mg/kg	$\mathrm{Increase}\;\mathrm{in}\;\mathrm{Rat}\;\mathrm{Paw}\;\mathrm{Volume},\;u.o.,\;\bar{x}\pm \Delta \bar{x}$, *n* = 8/ Inflammatory Response Inhibition, %		
			1 h	2 h	4 h
I	AR1	100	43.80 ± 0.07 *	58.43 ± 0.04	82.34 ± 0.08 **
			2.82	5.8	9.84
II	AR2	100	42.88 ± 0.04 *	56.45 ± 0.02 **	45.52 ± 0.03
			6.8	15.7	20.52
III	AR3	100	49.03 ± 0.03	62.46 ± 0.03	65.35 ± 0.04
			4.7	6.3	11.8
IV	Quercetin	50	46.57 ± 0.19	63.2 ± 0.15	61.09 ± 0.15
			5.8	13.4	17.2
V	Diclofenac sodium	8	39.17 ± 0.93 **	33.60 ± 0.05 *	35.52 ± 0.03 *
			17.97	31.54	45.14
VI	Control	-	44.88 ± 0.04	71.67 ± 0.02	132.29 ± 0.04

Notes: *—statistically significant difference compared to the control pathology group (*p* ≤ 0.05); **—statistically significant difference compared to the comparison drug diclofenac sodium (*p* ≤ 0.05). AR1—the soft extract from *A. reptans* L. herb (extractant water); AR2—the soft extract from *A. reptans* L. herb (extractant 50% ethanol); AR3—the soft extract from *A. reptans* L. herb (extractant 70% ethanol).

**Table 6:** The effect of *Ajuga reptans* L. herb extracts and the reference preparation “Rotokan” on the duration of bleeding.

№	Tested Substance	$\mathrm{Bleeding}\;\mathrm{Duration}\;\mathrm{Time}\bar{x}\pm \Delta \bar{x}$, c	Reduction in Bleeding Time, %
1	AR1	90.45 ± 1.88	40.59
2	AR2	110.35 ± 1.12	27.52
3	AR3	125.10 ± 1.44	17.83
4	«Rotokan»	72.15 ± 1.11	52.61
5	Intact animals	152.25 ± 1.65	-

Notes: AR1—the soft extract from *A. reptans* L. herb (extractant water); AR2—the soft extract from *A. reptans* L. herb (extractant 50% ethanol); AR3—the soft extract from *A. reptans* L. herb (extractant 70% ethanol).

**Table 7:** Dynamics of the wound healing process with the application of *Ajuga reptans* L. herb extracts.

Experimental Animal Groups	Determination of the Wound Healing Area as a Percentage of the Total Wound Surface Area, %					
	3 Day	5 Day	7 Day	9 Day	14 Day	16 Day
AR1	4.30 ± 0.12	10.45 ± 0.10	46.90 ± 0.13	74.25 ± 0.21	92.80 ± 0.13	100
AR2	7.00 ± 0.16	25.83 ± 0.13	62.92 ± 0.14	100	100	100
AR3	6.75 ± 0.13	24.75 ± 0.12	60.44 ± 0.12	83.63 ± 0.12	100	100
«Rotokan»	7.50 ± 0.12	28.35 ± 0.12	69.45 ± 0.17	100	100	100
Intact animals	4.20 ± 0.16	19.15 ± 0.11	55.25 ± 0.14	70.14 ± 0.14	92.24 ± 0.11	100

Notes: AR1—the soft extract from *A. reptans* L. herb (extractant water); AR2—the soft extract from *A. reptans* L. herb (extractant 50% ethanol); AR3—the soft extract from *A. reptans* L. herb (extractant 70% ethanol).

**Table 8:** Antimicrobial activity of the *Ajuga reptans* L. herb extracts.

Microorganism	Origin	Diameters of Growth Inhibition Zones, mm				
		Gentamicin	Fluconazole	*Ajuga reptans* L. Herb Extracts		
				AR1	AR2	AR3
*Staphylococci*						
*Staphylococcus (S.) aureus*	pus from a wound	6	0	0	0	6.36 ± 0.28
*S. haemolyticus*	pus from a boil	10	0	6.58 ± 0.12	6.95 ± 0.46	5.84 ± 0.27
*S. epidermidis*	ATCC	28	0	0	5.01 ± 0.64	4.48 ± 0.30
*Enterococci*						
*Enterococcus faecalis*	urethra	6	0	3.83 ± 0.20	7.29 ± 0.34 *	11.49 ± 0.61 *
*β-Hemolytic streptococci*						
*Streptococcus (St.) pyogenes (Group A)*	pharynx	8	0	0	11.96 ± 0.67 *	13.99 ± 0.67 *
*St. agalacticae (Group B)*	vaginal mucus	6	0	0	0	9.31 ± 0.49 *
*S. dysgalactiae* ssp. *equisimilis (group G)*	pharynx	8	0	4.39 ± 0.09	4.65 ± 0.26	12.27 ± 0.10 *
*α-Hemolytic streptococci*						
*St. gordonii*	oral cavity	6	0	4.63 ± 0.12	4.51 ± 0.30	13.24 ± 0.60 *
*St. sanguinis*	oral cavity	6	0	0	11.82 ± 0.40 *	0
*St. oralis*	oral cavity	6	0	[8.96 ± 0.61] *	12.14 ± 0.34 *	12.13 ± 0.13
*St. pneumoniae*	mucus	6	0	[6.37 ± 0.24] *	[10.86 ± 0.30] *	[11.01 ± 1.57] *
*Bacilli*						
*Bacillus subtilis*	ATCC 6051	34	0	4.07 ± 0.29	8.29 ± 0.48 *	4.48 ± 0.69
*Enterobacteria*						
*Escherichia coli*	ATCC 25922	22	0	0	0	5.80 ± 0.42
*Escherichia fergusonii*	defecation	12	0	0	0	3.86 ± 0.19
*Citrobacter freundii*	defecation	8	0	0	3.89 ± 0.08	12.45 ± 0.50 *
*Morganella morganii*	urine	6	0	4.07 ± 0.14	4.44 ± 0.35	5.66 ± 0.10 *
*Providencia rettgeri*	urine	6	0	0	0	9.09 ± 0.21 *
*Providencia stuartii*	urine	6	0	0	5.10 ± 0.44	8.61 ± 0.46 *
*Klebsiella pneumoniae*	ATCC 700603	12	0	0	0	5.68 ± 0.40
*Pseudomonads*						
*Pseudomonas aeruginosa*	pus from a wound	6	0	0	0	0
Yeast-like Fungi of the Genus *Candida*						
*Candida albicans*	oral cavity	0	6	0	0	[10.36 ± 0.48]
*C. tropicalis*	mucus	0	6	0	0	[5.35 ± 0.63]
*C. lusitaniae*	oral cavity	0	19	3.92 ± 0.16	5.25 ± 0.72	[4.98 ± 0.55]
*C. lipolytica*	oral cavity	0	6	0	4.65 ± 0.55	[5.83 ± 0.28]
*C. kefyr*	oral cavity	0	20	3.10 ± 0.16	4.38 ± 0.29	[9.55 ± 0.35]

Notes: [] Partial inhibition zones of microorganism growth (bacteriostatic/fungistatic effect) are shown in square brackets; *—*p* < 0.01 compared to the control (pure extraction agent). AR1—the soft extract from *A. reptans* L. herb (extractant water); AR2—the soft extract from *A. reptans* L. herb (extractant 50% ethanol); AR3—the soft extract from *A. reptans* L. herb (extractant 70% ethanol).

## Data Availability

The data supporting the results of this study can be obtained from the corresponding authors upon reasonable request.

## Supplementary Materials

The following supporting information can be downloaded at: https://www.mdpi.com/article/10.3390/plants14020219/s1. The assay methods are presented in the file Figure S1 and Methods S1.
