# Supplementary material for: Ajuga reptans L. Herb Extracts: Phytochemical Composition and Pharmacological Activity Screening
Source: Plants (Basel). 2025 Jan 14;14(2):219. doi: 10.3390/plants14020219 (PMC11768386; doi:10.3390/plants14020219)
Supplement: Supplementary file 1 [file plants-14-00219-s001.zip › plants-3413338-supplementary.pdf]

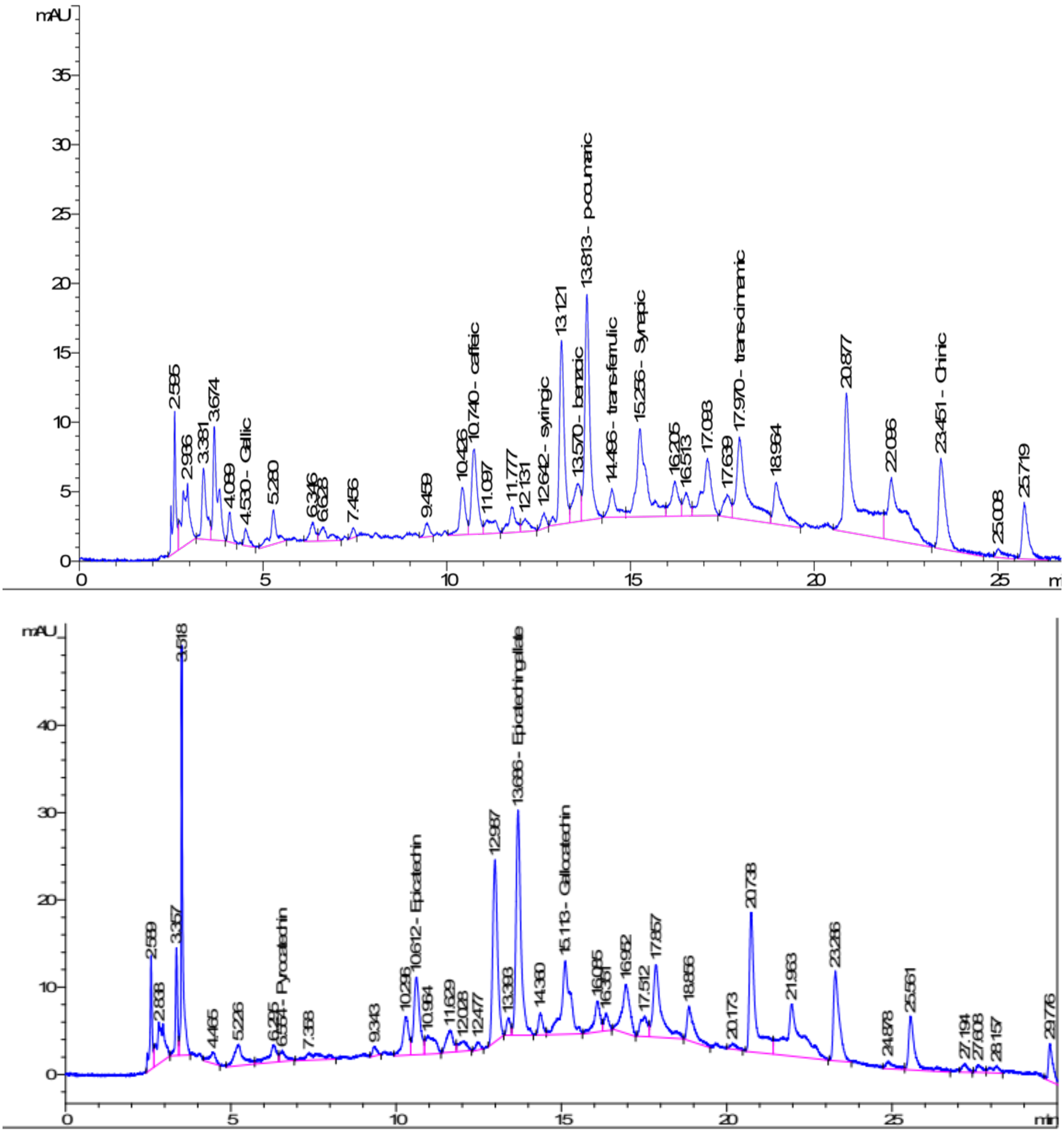

Figure S1. Typical HPLC chromatograms of phenolics in the extracts of *Ajuga reptans* L. herb

The quantitative determination of vitamin K content in the studied samples of *A. reptans* herb extracts

A sample of the *Ajuga reptans* extract (500 mg) was placed in a 50 mL conical flask and extracted three times with 70% ethanol, using 25 mL each time, by heating in a boiling water bath for 15 minutes. The extracts were filtered while hot into a 100 mL flask and then washed with 10 mL of 70% ethanol. 4 mL of a 10% lead acetate solution was added to the hot extract solutions and heated in a water bath for 3 minutes to coagulate the precipitate. The solution was then cooled to room temperature, filtered into a 100 mL volumetric flask, and brought to volume with 70% ethanol. A 5 mL portion of the resulting solution was transferred to a volumetric flask and diluted with the same solvent to 50 mL. The optical density of the extract was measured at a wavelength of 230 nm in a 10 mm cell using a spectrophotometer, with 70% ethanol serving as the reference solution. The optical density of a 10% Vikasol solution was measured in parallel. The vitamin K content (X) recalculated to Vikasol was determined using the formula:

$$X = \frac{A \times 100 \times 50 \times 100}{420 \times m \times 5 \times (100 - W)},$$

where A is the optical density of the test solution; m is the weight of the medicinal plant raw material (g); 420 is the specific absorption coefficient of Vikasol in 70% ethanol; and W is the mass loss on drying (%).

The content of free organic acids, recalculated as malic acid, in the extracts.

The content of free organic acids in the *Ajuga reptans* extract was determined by titration according to the pharmacopoeial method.

500 mg (accurate weight) of the *Ajuga reptans* extract are placed into a 250 mL flask, 200 mL of water is added, and the mixture is heated for 2 hours in a water bath. The solution is then cooled, quantitatively transferred to a 250 mL volumetric flask, the volume is brought up to the mark, and the solution is mixed thoroughly. From the prepared extract, 10 mL is transferred to a 500 mL flask. To this, 200–300 mL of freshly boiled water, 2 drops of 1% alcoholic phenolphthalein solution, and 1 drop of 0.1% methylene blue solution are added. The mixture is titrated with sodium hydroxide solution (0.1 mol/L) until a lavender-red coloration appears in the foam.

The content of free organic acids, recalculated to malic acid in absolutely dry raw material (X), in percentage, was calculated using formula:

$$X = \frac{V \times 0.0067 \times 250 \times 100 \times 100}{m \times 10 \times (100 - W)},$$

where: 0.0067 – the amount of malic acid corresponding to 1 mL of sodium hydroxide solution (0.1 mol/L), in grams; V – the volume of sodium hydroxide solution (0.1 mol/L) used for titration, in mL; m – s the mass of the extract, in grams; w – the moisture loss of the extract upon drying, in %.

The quantitative determination of ascorbic acid.

An analytical sample (2,0) of the *Ajuga reptans* extract was placed in a flask and 300 mL of purified water was added. The resulting mixture was infused for 10 minutes, stirred, and filtered. The analysis was then performed according to the pharmacopoeial method. In a conical flask, 1 mL of the obtained filtrate is added, along with 1 mL of a 2% hydrochloric acid solution and 13 mL of distilled water. The mixture is stirred and titrated with a microburette using a sodium 2,6-dichlorophenolindophenolate solution (0.001 mol/L) until a pink coloration appears that persists for 30–60 seconds. The titration should not exceed 2 minutes. The content of ascorbic acid, recalculated to absolute dry raw material in percentage terms, was calculated using formula:

$$X = \frac{V \times 0{,}000088 \times 300 \times 100 \times 100}{m \times 1\ (100 - w)},$$

where V – the volume of sodium 2,6-dichlorophenolindophenolate solution (0.001 mol/L) used for titration, in mL; 0.000088 is the amount of ascorbic acid corresponding to 1 mL of sodium 2,6-dichlorophenolindophenolate solution (0.001 mol/L), in grams; m - the weight of the tested raw material sample, in grams; w - the moisture loss of the extract upon drying, in %.

The quantitative determination of total polyphenols and tannins, expressed as pyrogallol.

0.100 g of the *Ajuga reptans* extract is placed in a round-bottom flask with a capacity of 250 mL and the volume of the solution is brought to 250 mL with water *R*. After that the solution is filtered through paper filter. Discard the first 25 mL of filtrate (solution A).

Total polyphenols. Dilute 5.0 ml of the filtrate to 25.0 mL with water *R*. Mix 2.0 ml of this solution with 1.0 mL of phosphomolybdotungstic reagent *R* and 10.0 ml of water *R* and dilute to 25.0 ml with a 290 g/L solution of sodium carbonate *R*. After 30 min measure the absorbance (2.2.25) at 760 nm (A1), using water *R* as the compensation liquid.
Polyphenols not adsorbed by hide powder. To 10.0 mL of the fltrate, add 0.10 g of hide powder CRS and shake vigorously for 60 min. Filter and dilute 5.0 mL of the filtrate to 25.0 mL with water *R*. Mix 2.0 mL of this solution with 1.0 mL of phosphomolybdotungstic reagent *R* and 10.0 mL of water *R* and dilute to 25.0 mL with a 290 g/L solution of sodium carbonate *R*. After 30 min measure the absorbance (2.2.25) at 760 nm (A2), using water *R* as the compensation liquid.

Standard solution. Immediately before the using, 50.0 mg of pyrogallol *R* is dissolved in water *R* and the volume of the solution is adjusted to 100.0 mL with the same solvent. 5.0 mL of the resulting solution is brought to a volume of 100.0 mL with water *R*.

A mixture of 2.0 mL of the resulting solution, 1.0 ml of phosphoromolybdenum tungsten reagent *R* and 10.0 ml of water *R* is brought to a volume of 25.0 mL with a solution of 290 g/L sodium carbonate *R*. After 30 minutes, measure the optical density of the solution at a wavelength of 760 nm (A3), using water *R* as a compensating solution.

The content of polyphenols, in terms of pyrogallol, in percent, is calculated by the formula:

$$X = \frac{62.5 \times A1 \times m2}{A3 \times m1},$$

where m1 is the mass of the tested sample, g; m2 is the mass of pyrogallol, g.

The content of tannins, in terms of pyrogallol, in percent, is calculated by the formula:

$$X = \frac{62.5 \times (A1 - A2) \times m2}{A3 \times m1},$$

where m1 is the mass of the tested sample, g; m2 is the mass of pyrogallol, g.

The quantitative determination of the total flavonoid content

About 0.25 g of the extract (exact weight) was introduced into a measuring flask with a capacity of 25.0 mL, dissolved in 70% ethyl alcohol with stirring, the volume of the solution in the flask was brought up to the mark with the same solvent and mixed (solution B).

2.0 mL of solution B was placed into a 25 mL volumetric flask, 2.0 mL of 3% aluminum chloride in 96% ethyl alcohol was added, the volume was brought up to the mark with 70% alcohol and mixed. After 30 min, the solution was filtered through a paper filter, discarding the first portions of the filtrate, and the optical density of the obtained complex was measured on a spectrophotometer at a wavelength of 417 nm in a cuvette with a layer thickness of 10 mm. The reference solution was a solution containing 2.0 ml of solution B, made up to the mark with 70% ethyl alcohol in a 25.0 ml volumetric flask.

In parallel, under the same conditions, an experiment was conducted with a solution of standard rutin.

1.0 mL of 3% alcohol solution of aluminum chloride was added to 1.0 mL of standard solution and diluted to 25.0 mL with 70% alcohol. As a comparison solution, a solution of standard rutin was used, brought up to the mark with 70% ethyl alcohol in a 25.0 mL volumetric flask.

The content of flavonoids in the research objects in terms of rutin was calculated as a percentage according to the formula:

$$X = \frac{A1 \times a0 \times 25 \times 1 \times 25 \times 100 \times 100}{A0 \times a1 \times 25 \times 2 \times 25 \times (100 - w)},$$

where A1 is the optical density of the solution under investigation; A0 is the optical density of the solution of the standard rutin complex with aluminum chloride; a1 – weight of the extract, g; a0 - the weight of the standard routine, g; w – mass loss during drying, %.

Preparation of standard solution is routine. About 0.01 g (exactly weighed) of rutin (FS 42-2508-87), dried at a temperature of 135 °C to a constant mass, was introduced into a volumetric flask with a capacity of 25 mL, dissolved in 96% alcohol, the volume of the solution was adjusted to the mark and stirred.

Preparation of a 3% solution of aluminum chloride in 96% alcohol. 3 g of aluminum chloride (DST 3759-85) was dissolved in 50 mL of 96% alcohol in a volumetric flask with a capacity of 100 mL, the volume of the solution was brought up to the mark with the same solvent and mixed.

The quantitative determination of hydroxycinnamic acids in terms of chlorogenic acid equivalents.

The content of hydroxycinnamic acid derivatives in the extracts was determined by the spectrophotometric method in terms of chlorogenic acid.

0.1 g (precise weight) of the extract was dissolved in a flask with a capacity of 100 mL, the volume of the solution was brought up to the mark with alcohol (50% v/v) *R* and mixed. The resulting solution is filtered through a paper filter, discarding the first 15 mL of filtrate (solution C).

Test solution: 1.0 mL of solution C is placed in a volumetric flask with a capacity of 10 mL, successively added, stirring after each addition, 2 mL of a 0.5 M solution of hydrochloric acid, 2 mL of a freshly prepared solution of 10 g of sodium nitrite *R* and 10 g of sodium molybdate R in 100 mL of water R, 2 mL of diluted sodium hydroxide solution *R*, bring the volume of the solution up to the mark with water *R* and mix.

Compensation solution. 1.0 mL of the original solution is mixed in a volumetric flask with a capacity of 10 mL, successively added, stirring after each addition, 2 mL of a 0.5 M solution of hydrochloric acid and 2 mL of a diluted sodium hydroxide solution *R*, the volume of the solution is brought up to the mark with water *R* and mixed. Immediately measure the optical density of the test solution at a wavelength of 525 nm in a cuvette with a layer thickness of 10 mm, using a compensating solution as a comparison solution.

The content of the sum of hydroxycinnamic acids, in terms of chlorogenic acid, in percent, is calculated according to the formula:

$$X = \frac{A \times 1000}{188 \times m},$$

where A is the optical density of the tested solution at a wavelength of 525 nm; m is the weight of the tested extract, g. The specific absorption index of chlorogenic acid is used, which is equal to 188.
